# Genetic and Structural Data on the SARS-CoV-2 Omicron BQ.1 Variant Reveal Its Low Potential for Epidemiological Expansion

**DOI:** 10.3390/ijms232315264

**Published:** 2022-12-03

**Authors:** Fabio Scarpa, Daria Sanna, Domenico Benvenuto, Alessandra Borsetti, Ilenia Azzena, Marco Casu, Pier Luigi Fiori, Marta Giovanetti, Antonello Maruotti, Giancarlo Ceccarelli, Arnaldo Caruso, Francesca Caccuri, Roberto Cauda, Antonio Cassone, Stefano Pascarella, Massimo Ciccozzi

**Affiliations:** 1 Department of Biomedical Sciences, University of Sassari, 07100 Sassari, Italy; 2 Unit of Medical Statistics and Molecular Epidemiology, University Campus Bio-Medico of Rome, 00128 Rome, Italy; 3 National HIV/AIDS Research Center, Istituto Superiore di Sanità, 00161 Rome, Italy; 4 Department of Veterinary Medicine, University of Sassari, 07100 Sassari, Italy; 5 Flavivirus Laboratory, Oswaldo Cruz Institute, FIOCRUZ, Rio de Janeiro 21040-900, Brazil; 6 Department of Science and Technology for Humans and the Environment, University of Campus Bio-Medico di Roma, 00128 Rome, Italy; 7 Department GEPLI, Libera Università Ss Maria Assunta, 00193 Rome, Italy; 8 Department of Public Health and Infectious Diseases, University Hospital Policlinico Umberto I, Sapienza University of Rome, 00161 Rome, Italy; 9 Department of Molecular and Translational Medicine, Section of Microbiology, University of Brescia, 25123 Brescia, Italy; 10 UOC Malattie Infettive, Infectious Disease Department, Fondazione Policlinico Universitario Agostino Gemelli IRCCS, 00168 Rome, Italy; 11 Center of Genomic, Genetic and Biology, 53100 Siena, Italy; 12 Department of Biochemical Sciences “A Rossi Fanelli”, Sapienza Università di Roma, 00161 Rome, Italy

**Keywords:** coronavirus, SARS coronavirus, epidemiology, pandemics, virus classification, BQ.1

## Abstract

The BQ.1 SARS-CoV-2 variant, also known as Cerberus, is one of the most recent Omicron descendant lineages. Compared to its direct progenitor BA.5, BQ.1 has some additional spike mutations in some key antigenic sites, which confer further immune escape ability over other circulating lineages. In such a context, here, we perform a genome-based survey aimed at obtaining a complete-as-possible nuance of this rapidly evolving Omicron subvariant. Genetic data suggest that BQ.1 represents an evolutionary blind background, lacking the rapid diversification that is typical of a dangerous lineage. Indeed, the evolutionary rate of BQ.1 is very similar to that of BA.5 (7.6 × 10^−4^ and 7 × 10^−4^ subs/site/year, respectively), which has been circulating for several months. The Bayesian Skyline Plot reconstruction indicates a low level of genetic variability, suggesting that the peak was reached around 3 September 2022. Concerning the affinity for ACE2, structure analyses (also performed by comparing the properties of BQ.1 and BA.5 RBD) indicate that the impact of the BQ.1 mutations may be modest. Likewise, immunoinformatic analyses showed moderate differences between the BQ.1 and BA5 potential B-cell epitopes. In conclusion, genetic and structural analyses on SARS-CoV-2 BQ.1 suggest no evidence of a particularly dangerous or high expansion capability. Genome-based monitoring must continue uninterrupted for a better understanding of its descendants and all other lineages.

## 1. Introduction

To date, the world continues its fight against the COVID-19 pandemic, declared by the World Health Organization (WHO) on 11 March 2019 (https://www.who.int/director-general/speeches/detail/who-director-general-s-opening-remarks-at-the-media-briefing-on-covid-19, accessed on 9 November 2022). As of 6 November 2022, the WHO stated that there were over 629 million confirmed cases and over 6.5 million deaths due to COVID-19 (https://www.who.int/publications/m/item/weekly-epidemiological-update-on-covid-19, accessed on 9 November 2022). This pandemic has been caused by the coronavirus SARS-CoV-2, which was identified for the first time in December 2019 during a pneumonia outbreak in Wuhan (China) [1]. Along the pandemic course, SARS-CoV-2 evolved, with the generation of many new variants endowed with a progressive increase in transmissibility [2], hence perpetuating the pandemic despite the availability of safe and effective vaccines since early 2021. With time, the virus variants have shown an increasing trend of escaping from the antibody immunity conferred by the vaccines (https://www.who.int/health-topics/vaccines-and-immunization#tab=tab_1, accessed on 5 November 2022), and infections are likely to remain a problem for the time being in most countries. SARS-CoV-2 is a positive-sense, single-stranded RNA virus, of which replication and transcription are made by the viral RNA-dependent RNA polymerase (RdRp, nsp12) [3]; during its RNA replication, very often, errors occur [4]. Owing to this rapid evolution, mainly shaped by natural selection, the formation of new variants is not a novelty or sporadic case but a certainty that periodically recurs [2,5]. Accordingly, many variants have emerged since the pandemic started in China and have continued to emerge up to the present day. Some of these variants have been given Greek names (i.e., Alpha, Beta, Gamma, Delta, and Omicron) and declared by the WHO to be of particular concern because of their high transmissibility and impact on populations’ health (https://www.who.int/news/item/31-05-2021-who-announces-simple-easy-to-say-labels-for-sars-cov-2-variants-of-interest-and-concern, accessed on 5 November 2022). Since the end of 2021, the Omicron variant has out-competed previous variants, becoming the dominant variant worldwide and it is continuing to spread through the generation of a number of subvariants, which include BA.1, BA.2, BA.3, BA.4, BA.5, and their descendant lineages (https://www.who.int/activities/tracking-SARS-CoV-2-variants, accessed on 5 November 2022).

One of the most recent descendant lineages is represented by the Omicron subvariant BQ.1, also known as Cerberus [6]. BQ.1 is a direct descendant of BA.5, with additional spike mutations in some key antigenic sites (K444T and N460K). Its first descendant, BQ.1.1 (as of 28 November 2022, there are at least nine further direct descendants), carries a further additional mutation (R346T) [7]. As of 9 October 2022, BQ.1 has been detected in 65 countries, with an overall prevalence of 6% (https://www.who.int/news/item/27-10-2022-tag-ve-statement-on-omicron-sublineages-bq.1-and-xbb, accessed on 5 November 2022). Considering that these additional mutations confer further immune escape ability over other circulating lineages [7,8], constant genome-based monitoring is mandatory.

In such a context, we have here devised an approach whereby results on genetic variability/phylodynamics, structural, and immunoinformatics are compared and integrated in order to obtain a complete-as-possible nuance of this rapidly evolving and potentially high transmissible Omicron subvariant. More specifically, the objective of our research is to gather data that can validate or argue against the concern of a worldwide epidemiological expansion of such a strongly immune-evasive variant as the Omicron BQ.1. 

## 2. Results

**Figure 1:**
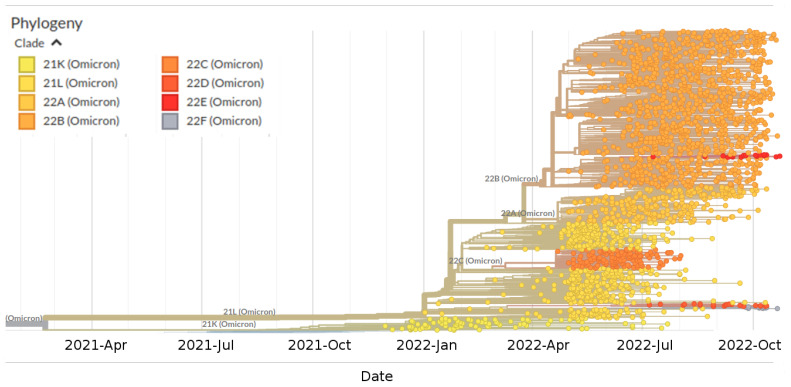
Highlight of the Omicron clade (GSAID Clade 21L) in the time-scaled phylogenetic tree of a representative global subsample of 2809 SARS-CoV-2 genomes sampled between January 2022 and November 2022. Phylogeny has been reconstructed using nextstrain/ncov (https://github.com/nextstrain/ncov, accessed on 9 November 2022), available at https://gisaid.org/phylodynamics/global/nextstrain/, accessed on 9 November 2022. The figure has been edited using the software GIMP 2.8 (available at https://www.gimp.org/downloads/oldstable/, accessed on 9 November 2022). See Table 1 for details on Nextstrain clade, Pango lineage and WHO labels.

**Figure 2:**
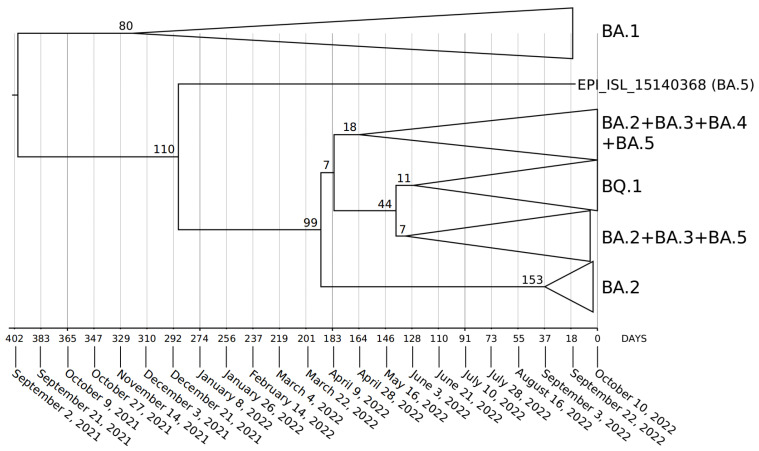
Maximum clade credibility tree estimated from 1575 whole genomes of BQ.1, BA.1, BA.2, BA.3, BA.4, and BA.5 downloaded from the GSAID portal (https://gisaid.org/), available on 15 October 2022. See Table S1 for details on the genomes included in the analyses. All showed nodes are well-supported, and the values of posterior probabilities for all the nodes are between 0.95 and 1 (PP ≥ 0.95). The bar under the tree indicates the time scale expressed in days before 10 October 2022, which represents the most recent sampling date included in the analyzed dataset. Node values indicate branches time expressed in days.

**Figure 3:**
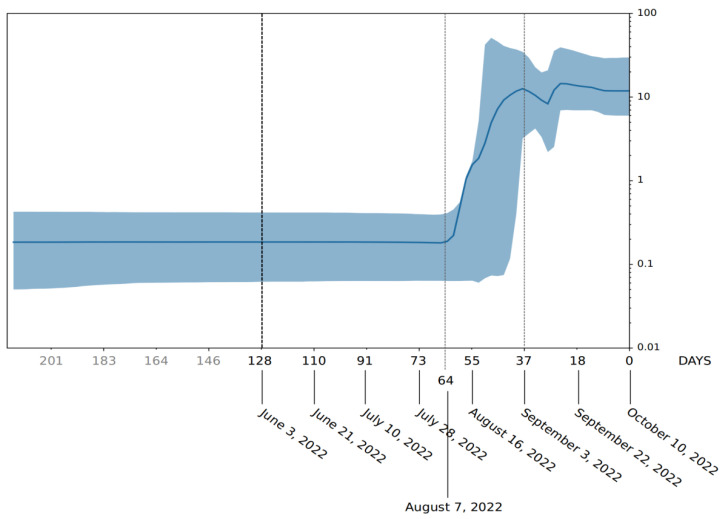
Bayesian skyline plot of SARS-CoV-2 BQ.1 variant. The viral effective population size (y-axis) is shown as a function of days (x-axis). The solid area represents the 95% high posterior density (HPD) region.

**Figure 4:**
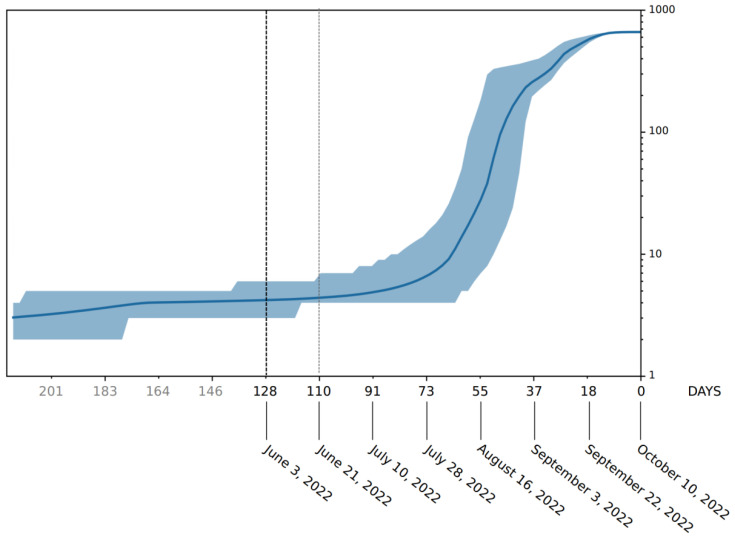
SARS-CoV-2 BQ.1 variant lineages through time. The number of lineages (y-axis) is shown as a function of days (x-axis). The solid area represents the 95% high posterior density (HPD) region.

Phylogenomic reconstruction (Figure 1) indicates that the genomes of BQ.1 (GSAID Clade 22E) are clustered within the not-monophyletic GSAID Clade 21L, with a close relationship with its direct progenitor BA.5 (See Table 1 for details on Nextstrain clade, Pango lineage and WHO labels). Results of the Bayes factor on the dataset of 1575 genomes revealed that the Bayesian skyline model under the log-normal uncorrelated relaxed clock model fitted data significantly better than other tested demographic and clock models. The maximum clade credibility tree (Figure 2) indicates that all genomes of BQ.1 are clustered together in a monophyletic group whose common ancestor was temporally placed 128 days before 10 October 2022 (i.e., 3 June 2022). The Bayesian skyline plot (BSP) (Figure 3) shows that the viral population underwent an increase in size starting from 64 days before 10 October 2022 (i.e., 7 August 2022), reaching the peak at about 37 days before 10 October 2022 (i.e., 3 September 2022). The lineages through time plot (Figure 4) indicates that the increase in the number of lineages started about 110 days before 10 October 2022 (i.e., 21 June 2022). The evolutionary rate, co-estimated with BSP and the lineages through time, amounts to 7.6 × 10^−4^ [95% HPD 5.2 × 10^−4^–9.8 × 10^−4^] subs/sites/years.

**Figure 5:**
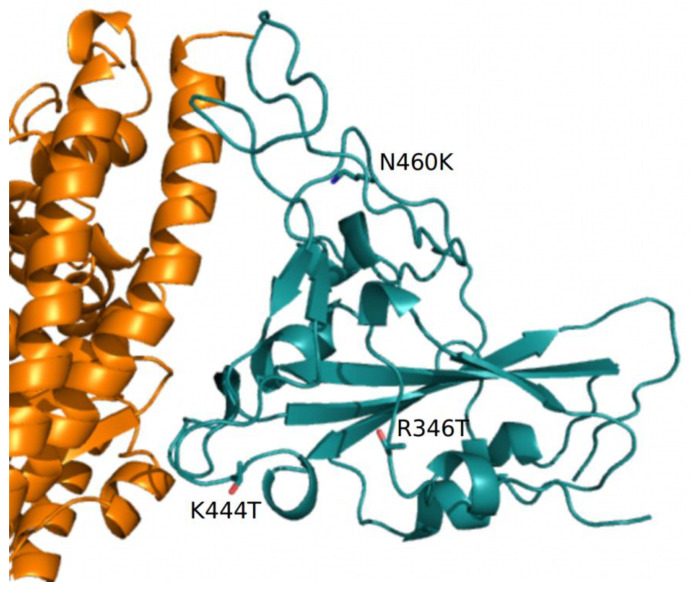
Complex between ACE2 (orange) and RBD (deep teal) represented as cartoon models. The side chains of the residue mutations specific to BQ.1 are displayed with stick models and are labeled. Only part of the ACE2 receptor is displayed.

The predicted structural properties of the BQ.1 spike have been compared with those of the BA.5 spike. The characterizing BQ.1 mutations are reported in Table S2. The N-terminal domains (NTDs) of the two spikes are identical, while the receptor binding domains differ for the three BQ.1 mutations R346T, K444T, and N460K. N460K has also been reported in BA.2.75. Homology modeling of the BQ.1 spike indicates that the R346T mutation occurs in a loop exposed to the solvent, while K444T is inside the receptor binding motif near the interface to ACE2 (Figure 5). N460K also occurs in an exposed loop not directly involved in the interaction with ACE2 (Figure 5). 

**Figure 6:**
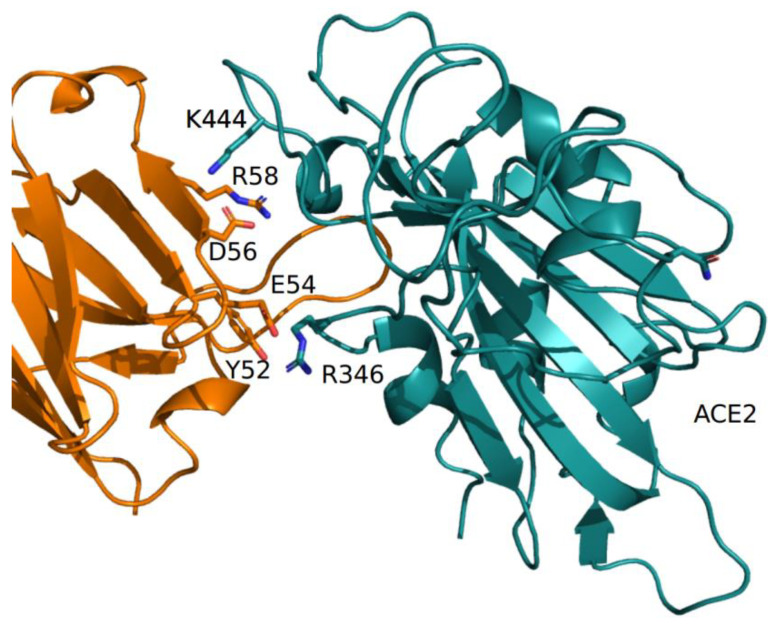
Complex between the COV38-142 antibody-heavy chain (orange) and RBD (deep teal), as reported in the PDB coordinate set 7LM8. Interacting side chains are displayed with stick models and are labeled. The label «ACE2» marks the interface to the receptor.

The predicted net charge of the RBD at pH = 7.0 (set as a reference pH, though not necessarily reflecting the physiological environment) was calculated by means of the program PROPKA3. The predicted overall net charge is dependent on the combination of the sidechain charges. The net charge is influenced by the interactions of the side chains with the surrounding atoms. To sample the variability of the interactions, 100 homology models of each RBD were calculated with Modeller. Indeed, the Modeller refinement stage of homology modeling can produce models differing in the conformation of side chains. Each model was optimized using the Foldx5 “RepairPDB” procedure, and the net charge was calculated by PROPKA3. The final charge was the average of the 100 charges, and the variability was estimated by standard error at a 95% confidence interval. The same procedure was applied to estimate the interaction energy of the complex ACE2-RBD. The net charge attributed to the BA.5 and BQ.1 identical NTD spike moieties is +1.18 ± 0.09. The BA.5 and BQ.1 RBD shows a net charge equal to +5.18 ± 0.03 and +4.20 ± 0.03, respectively. The decreased charge is coherent with the replacement of two positively charged residues, R346 and K444, by the uncharged polar Thr. The pattern was confirmed by the comparison of the electrostatic potential surfaces of the two RBDs. The surface of the BA.5 RBD has a more positively charged region with respect to the corresponding surface in BQ.1 (see Figure S1). According to visual and Foldx5 interface analyses, Thr444 does not directly interact with ACE2 (Figure 5). Interaction energies between ACE2 and BA.5 and BQ.1, estimated by Foldx5, were −4.67 ± 0.55 and −4.50 ± 0.57 Kcal/mol, respectively. The interaction between ACE2 and BQ.1 was predicted to be slightly weaker than in the case of BA.5 within the limits of the method. Even if the influence of the characterizing mutations of BQ.1 RBD appears marginal on the interaction with ACE2, they may have an impact on the interaction with antibodies. As an example, the interaction with the monoclonal antibody CV38-192, as reported in the PDB data set 7LM8, has been examined. The RBD positions 346 and 444 are at the interface with the antibody-heavy chain (Figure 6). 

Foldx5 analysis suggests that in the wild-type RBD, the residue R346 interacts with E54 and Y52 while K444 interacts with D56 of the heavy chain. Removal of the positive charges of R346 and K444 should disrupt the salt bridges. In silico mutagenesis was applied to replace the single position and both positions with threonine, as in BQ.1. Interaction energies were predicted by Foldx5. The results suggest that the mutations tend to destabilize the interaction with the antibody-heavy chain (Table 2). Moreover, the analysis by DrugScore (PPI) confirms that R346 is a major hotspot. Indeed, the loss of interaction energy of the complex upon the mutation of R346 to alanine is rather high and equal to G = 1.82 Kcal/mol.

The immunoinformatic analysis found the presence of 108 B-cell epitope residues in the BQ.1 strain and 111 B-cell epitope residues in the BA.5 strain of the K439L mutation of BQ.1. The main differences between BA.5 and BQ.1 are reported in Table 3.

## 3. Discussion

The SARS-CoV Omicron BQ.1 variant represents one of the most recently discovered lineages, and it requires an in-depth study on its capacity for expansion and contagiousness. Here, we sought a deep insight into the evolutionary and structural patterns of the SARS-CoV BQ.1 variant by means of all genomes available in GSAID on 15 October 2022. 

Phylogenomic reconstruction indicates that genomes of BQ.1 (GSAID Clade 22E) are clustered within the wide GSAID Clade 22B (BA.5). This is not surprising, considering that BQ.1 is a descendant of BA.5. One of the most striking results is given by the evolutionary condition of BQ.1; indeed, the phylogeny suggests an evolutionary condition very similar to the variants BA.2.75 (GSAID Clade 22D) and BA.2.12.1 (GSAID Clade 22C), which represent an evolutionary blind background with no further epidemiologically relevant descendant. If this evolutionary condition is confirmed, it will be possible to appreciate in BQ.1 and its descendant an atypical accumulation of neutral loss-of-function mutations. Indeed, these kinds of lineages are typically characterized by several nucleotidic mutations with no (or very few) amino acidic mutations for which important genes are concerned. In addition, the branches’ length in the phylogenetic tree suggests a lack of rapid diversification, which is typical of a dangerous lineage at the beginning of its evolutionary path. 

Phylodynamic reconstruction performed on a dataset comprising 1575 genomes indicated that among the included variants (BQ.1, BA.1, BA.2, BA.3, BA.4, BA.5 and their descendant sublineages), BA.1 and BQ.1 are the only ones to show a monophyletic condition. In the maximum clade credibility tree, the common ancestor to all genomes of BQ.1 was temporally placed 127 days before 10 October 2022 (which is the most recent collection date), i.e., 3 June 2022. This dating predates by about one month the first detected genome of BQ.1 (for which a complete sampling date is available), which was isolated in Nigeria (Abuja) on 4 July and initially labeled as BA.5-like (and then relabeled as BQ.1). It is interesting to note that considering its date of origin, the virus circulated undisturbed for a long time before being detected. This is not the feature of a dangerous variant, which typically explodes much faster in terms of the number of infections and population size. Accordingly, a Bayesian skyline plot (BSP) reconstruction, estimated on 1114 whole genomes of BQ.1 (collection range 4 July 2022–10 October 2022), indicated a low level of genetic variability, also confirmed by a genetic distance matrix, where the largest value amounted to 0.731 (±0.002); it was found in few cases between genomes sampled three months later. Indeed, after an initial low and flattened level of genetic variability, around 7 August 2022 (64 days before 10 October 2022), the expansion of the viral population size started with a very steep curve that lasted 18 days, after which the peak was reached around 3 September 2022. The reconstruction of lineages through time indicated that the increase of the lineage’s numbers started about 10 days before the beginning of the increase in the population size, which is usual in this kind of case. During the plateau phase, genetic variability (and, accordingly, viral population size) fluctuates, with some rise and fall but nothing relevant, and currently, the viral population size appears to be stable and flattened. It should be pointed out that this is not the typical trend of a lineage that is about to explode in terms of population size and, accordingly, in terms of contagiousness, as shown at the beginning of the pandemic when variability increased very quickly, with a very vertical curve (see, i.a., Lai et al. [9]). On the contrary, this trend is very similar to what has been shown for the BA.2.75 variant, similar to BQ.1, which, in the beginning, aroused much concern but, after a deep genome-based survey, did not show evidence of a particularly dangerous or high expansion capability, being even slower than others [10]. Indeed, the BQ.1 situation is coherent with a scenario quite typical of an evolutionary lineage that presents new features in comparison to its direct progenitor (BA.5), but these new features, at the present stage, do not represent a further boost able to promote an abnormal expansion. In addition, the lack of lineage increases over time further confirms the lack of an enlargement in the number of haplotypes in recent times. The evolutionary rate estimated for BQ.1 amounts to 7.6 × 10^−4^ subs/site/year, with a very narrow range (5.2 × 10^−4^–9.8 × 10^−4^ subs/site/year). This further confirms the low level of genetic variation and the poor capability of demographic expansion; indeed, it is very similar to variant BA.5, with 7 × 10^−4^ subs/site/year, and variant BA.2.75, with 1.6 × 10^−4^ subs/site/year [10]. Concerning the comparison with BA.5, it should be remarked that this last variant has been circulating for several months and its current level of variability is lower than in the early stages. Accordingly, the evolutionary rate of BQ.1 should be greater if it were dangerous and had a highly contagious capability lineage. Indeed, at the beginning of the current pandemic, the evolutionary rate of the first lineage of SARS-CoV-2 was about 6.5810^−3^ subs/site/year [11], which means that in this case, the new variant presents a 10^−1^ slower factor than the Wuhan-Hu-1 variant.

The comparison of the properties of BQ.1 and BA.5 RBDs predicts that the impact of the BQ.1 mutations on the affinity for ACE2 may be modest, although it appears to be potentially destabilizing. In fact, the calculation of the RBD net charge, that is, an indirect measure of the dominant charge of the electrostatic potential surface, suggests that it is decreased in BQ.1. The value of the RBD net charge in BQ.1 is comparable to that of the Delta variant and would suggest a diminution rather than an increase in virus transmissibility compared to the parental Omicron BA.5 variant [12]. Interestingly, two of the characterizing mutations that remove the charged residues in the Wuhan virus, R346 and K444, have a clear destabilizing effect on the interaction between RBD and the class of antibodies that recognizes the epitope containing the two positions. It may be speculated that the current evolution of the virus is optimizing its immune escape ability rather than its affinity for the receptor. The limited immunoinformatic analysis performed here has shown the differences between the BQ.1 and BA5 potential B-cell epitopes. Although such a difference may appear to be modest, it adds to the already established mutations of BA.5 spike epitopes in explaining the elevated capability of the BQ.1 variant to escape neutralizing antibodies generated by vaccination or infection [8]. Besides antibody responses, particularly those neutralizing virus entry into host cells, the COVID-19 disease is under the control of T-cell-mediated immunity, particularly CD4+T and CD8+T lymphocytes, of which CD8+T lymphocytes are endowed with potent cytotoxicity against SARS-CoV-2-infected cells [13]. In this immunological scenario, it may be of interest that the BQ1 R346T mutation may affect a T-cell-recognized immunogenic peptide that is largely preserved in all previous variants [14]. However, it is not known whether or to what extent the substitution of arginine by threonine will affect peptide immunogenicity. In addition, we have observed that all other highly preserved T-cells responsive to the epitopes of the spike protein, as reported by De la Fuente et al. [14], appear to be unmodified in the BQ.1 variant. Although further direct research on this topic is warranted, altogether, these data do not support any relevant immune evasion by BQ1 of the T-cell response against the above spike epitopes. 

In accordance with all evolutionary theories, often, new SARS-CoV-2 variants will exhibit antibody escape capabilities [15,16], but this is not necessarily strictly related to a high diffusion capability or to the improved pathogenicity of the virus [10]. According to the currently available data, SARS-CoV-2 BQ.1 appears as a new variant with no enhanced capabilities of infectivity or pathogenicity with respect to its direct progenitor BA.5. 

The original Omicron, named 21K and BA.1, according to Nextstrain clade and Pango lineage nomenclature, respectively (https://www.who.int/activities/tracking-SARS-CoV-2-variants, accessed on 5 November 2022), appears to have arisen in November 2021, probably in South Africa [17]. Indeed, early sequences were predominantly from South Africa (https://www.who.int/publications/m/item/weekly-epidemiological-update-on-covid-19, accessed on 5 November 2022) although the virus was almost simultaneously detected in Botswana and Hong Kong [17]. It is part of the large group 21M, which corresponds to the Pango lineage B.1.1.529. For all new variants, also for Omicron 1, the primary concern was represented by a large number of mutations in the spike gene, most of which were placed in the receptor binding domain and N-terminal domain and, thus, potentially able to play key roles in ACE2 binding and antibody recognition. Accordingly, Liu et al. [18], by testing monoclonal antibodies against all known epitope clusters on the spike protein for the lineage B.1.1.529, found that most of the tested antibodies were either abolished or impaired, with the occurrence of four new spike mutations that conferred greater antibody resistance on B.1.1.529. Conceivably, the original Omicron became worldwide dominant in a short time [18]. However, concerning the expansion capabilities of B.1.1.529, it should be pointed out that it is facilitated by the period; indeed, its major expansion occurred in the cold season, which is known to be favorable for infection by and transmission of respiratory viruses (see, i.a., Moriyama et al. [19]). The results presented for Cerberus are based on data mainly belonging to the warm season (4 July 2022–10 October 2022), and transmissibility and number of infections could undergo a variation during the cold season. However, it should be pointed that the plateau has been already reached, and new expansions, given current results, are more likely to be caused by its subvariants, which may accumulate new further mutations. 

## 4. Materials and Methods

The first genomic epidemiology of the SARS-CoV-2 BQ.1 Omicron variant (GSAID Clade 22E) was reconstructed by using a subsampling focused globally over the past 6 months, built with nextstrain/ncov (https://github.com/nextstrain/ncov, accessed on 9 November 2022), available at https://gisaid.org/phylodynamics/global/nextstrain/ (accessed on 9 November 2022), including all genomes belonging to the GSAID Clade 21 M (Omicron). The obtained tree was edited as an image using the software GIMP 2.8 (available at https://www.gimp.org/downloads/oldstable/, accessed on 9 November 2022). After the first genomic assessment, a subset of 1575 genomes was built up, including, together with all genomes of BQ.1 for which complete sampling data were available, representatives of the main variants of concern (BA.1, BA.2, BA.3, BA.4, BA.5). Genomes were aligned by using the algorithm L-INS-I implemented in Mafft 7.471 [20], producing a dataset that was 29,645 bp long. Manual editing was performed by using the software Unipro UGENE v.35 [21]. The software jModeltest 2.1.1 [22] was used to find the best probabilistic model of genome evolution with a maximum likelihood optimized search. The phylogenomic relationship among variants and time of divergence was investigated using Bayesian inference (BI), which was carried out using the software BEAST 1.10.4 [23], with runs of 200 million generations under several demographic and clock models. In order to obtain an inference on the best representative output, a selection of the better model was performed using the test of the Bayes factor [24] by comparing the 2lnBF of the marginal likelihood values following Mugosa et al. [25]. The phylogenetic trees were edited and visualized using FigTree 1.4.0 (available at http://tree.bio.ed.ac.uk/software/figtree/, accessed on 9 November 2022). The software Beast was also used to co-estimate the evolutionary rate, the Bayesian skyline plot (BSP), and lineages through time for the BQ.1 variant (with a subset of 1114 genomes), with runs of 300 million generations under the Bayesian skyline model with an uncorrelated log-normal relaxed clock model. All datasets were built by downloading genomes from the GSAID portal (https://gisaid.org/, accessed on 9 November 2022), available on 15 October 2022. See Table S1 for details on the genomes included in the dataset and authorship. 

Homology models of the mutant BQ.1 spikes were created by means of the software Modeller 10.3 [26]. Surface electrostatic potential was calculated and displayed with the graphic program PyMOL [27]. Net charges were calculated using the software PROPKA3 [28]. Foldx5 [29] was applied to optimize the side chain conformation of the models built by Modeller using the Foldx function “RepairPDB”. The interaction energy between the Spike RBD and ACE2 was predicted with the Foldx5 function “AnalyseComplex”. Interface residue–residue interactions were assessed with the Foldx5 “PrintNetwork” function. In silico mutagenesis was obtained with the built-in functions available within PyMOL. In silico alanine scanning of the residues at the interface between RBD and ACE2 was carried out using the method available via the web server DrugScore (PPI) [30], available at https://cpclab.uni-duesseldorf.de/dsppi/, accessed on 1 November 2022. The method is a fast and accurate computational approach to predict changes in binding free energy when each residue at the subunit interface is mutated into alanine. 

DiscoTope 2.0 was used to predict discontinuous B-cell epitopes from three-dimensional protein structures using the default DiscoTope score threshold of −3.700 [31].

## 5. Conclusions

In conclusion, genetic and structural analyses of SARS-CoV-2 BQ.1 suggest that, although this new variant presents several spike mutations of interest and overall highly immune-evasive neutralizing antibodies [8], currently, it does not show evidence of a particularly dangerous or high expansion capability. The Omicron variant of concern remains the dominant variant circulating globally (https://www.who.int/publications/m/item/weekly-epidemiological-update-on-covid-19---9-november-2022, accessed on 9 November 2022). BQ.1 expansion appears to be even slower than that of BA.5, which became dominant early 2022, and based on current data, it does not appear to present an alarming situation. However, this condition must not be understood as a reason to let down our guard against the pandemic or the generation of further variants. Indeed, new further mutations can make BQ.1 more dangerous or generate new subvariants. As of 8 November 2022, BQ.1 has presented four known sublineages, which appear to possess similar antibody escape capabilities. Accordingly, constant genome-based surveys remain the best tool for a better understanding of the phenomenon. The monitoring of BQ.1 and its descendants, as well as the monitoring of all other lineages, must continue uninterrupted in order to identify and/or predict important changes in genomic composition and/or diffusion capability.

## Figures and Tables

**Table 1:** Nextstrain clade, Pango lineage and WHO labels of the investigate lineages showed in Figure 1.

Nextstrain Clade	Pango Lineage	WHO Label
21K (Omicron)	BA.1	**o** (Omicron)
21L (Omicron)	BA.2	**o** (Omicron)
22A (Omicron)	BA.4	**o** (Omicron)
22B (Omicron)	BA.5	**o** (Omicron)
22C (Omicron)	BA.2.12.1	**o** (Omicron)
22D (Omicron)	BA.2.75	**o** (Omicron)
22E (Omicron)	BQ.1	**o** (Omicron)
22F (Omicron)	XBB	**o** (Omicron)

**Table 2:** Interaction energy between Wuhan and mutant RBDs and the CV38-142 antibody-heavy chain calculated by Foldx5.

RBD	Interaction Energy (Kcal/mol)
Wuhan	−6.3
Wuhan-R346T	−5.5
Wuhan-K444T	−6.1
Wuhan-R346T-K444T	−5.6

**Table 3:** The amino acidic position within the SARS-Cov-2 spike glycoprotein; the relative amino acids for BA.5 and BQ.1 are reported. DiscoTope scores for both variants are compared. Amino acids that have lost their potential to be considered probable B-cell epitopes are highlighted in red font and labeled with *.

BA5	Amino Acidic Position	BQ.1	BQ.1 DiscoTope Score	BA5 DiscoTope Score
SER	438	SER	−3.733 *	−2.003
LYS	439	THR	0.220	1.904
VAL	440	VAL	3.112	4.307
GLY	441	GLY	2.203	3.670
GLY	442	GLY	0.553	2.047
ASN	443	ASN	−3.125	−1.276
TYR	444	TYR	−1.383	0.031
ASN	445	ASN	−2.964	−1.719
LEU	487	LEU	−4.250 *	−3.559
GLN	488	GLN	−4.265 *	−3.436
SER	489	SER	−3.458	−2.349
TYR	490	TYR	−4.915 *	−3.658
GLY	491	GLY	−0.377	0.794
PHE	492	PHE	−3.202	−1.742
ARG	493	ARG	0.628	2.089
PRO	494	PRO	1.352	2.664
THR	495	THR	2.953	3.742
TYR	496	TYR	−0.290	0.704
GLY	497	GLY	1.939	2.529
VAL	498	VAL	−1.746	−0.966
GLY	499	GLY	−2.855	−2.103
HIS	500	HIS	−2.180	−1.223
GLN	501	GLN	−3.523	−2.327

## Data Availability

Genomes analyzed in the present study were taken from the GSAID database and are available at https://gisaid.org/ (accessed on 15 October 2022).

## Supplementary Materials

The following supporting information can be downloaded at: https://www.mdpi.com/article/10.3390/ijms232315264/s1.

## References

[1] Zhou P., Yang X.-L., Wang X.-G., Hu B., Zhang L., Zhang W., Si H.-R., Zhu Y., Li B., Huang C.-L. (2020). A pneumonia outbreak associated with a new coronavirus of probable bat origin. Nature.

[2] Zella D., Giovanetti M., Benedetti F., Unali F., Spoto S., Guarino M., Angeletti S., Ciccozzi M. (2021). The variants question: What is the problem?. J. Med. Virol..

[3] Hillen H.S., Kokic G., Farnung L., Dienemann C., Tegunov D., Cramer P. (2020). Structure of replicating SARS-CoV-2 polymerase. Nature.

[4] Scarpa F., Casu M., Sanna D. (2021). Evolutionary and Conservation Genetics. Life.

[5] Borsetti A., Scarpa F., Maruotti A., Divino F., Ceccarelli G., Giovanetti M., Ciccozzi M. (2022). The unresolved question on COVID-19 virus origin: The three cards game?. J. Med. Virol..

[6] Mustafa M., Makhawi A. (2022). What Learned from Omicron Sub-Variants BQ.1 and BQ.1.1. bioRxiv.

[7] Qu P., Evans J.P., Faraone J., Zheng Y.M., Carlin C., Anghelina M., Stevens P., Fernandez S., Jones D., Lozanski G. (2022). Distinct Neutralizing Antibody Escape of SARS-CoV-2 Omicron Subvariants BQ.1, BQ.1.1, BA.4.6, BF.7 and BA.2.75.2. bioRxiv.

[8] Kurhade C., Zou J., Xia H., Liu M., Chang H.C., Ren P., Xie X., Shi P.Y. (2022). Low neutralization of SARS-CoV-2 Omicron BA.2.75.2, BQ.1.1, and XBB.1 by 4 doses of parental mRNA vaccine or a BA.5-bivalent booster. bioRxiv.

[9] Lai A., Bergna A., Acciarri C., Galli M., Zehender G. (2020). Early phylogenetic estimate of the effective reproduction number of SARS-CoV-2. J. Med. Virol..

[10] Scarpa F., Sanna D., Azzena I., Giovanetti M., Benvenuto D., Angeletti S., Ceccarelli G., Pascarella S., Casu M., Fiori P.L. (2022). On the SARS-CoV-2 BA.2.75 variant: A genetic and structural point of view. J. Med. Virol..

[11] Benvenuto D., Giovanetti M., Salemi M., Prosperi M., De Flora C., Junior Alcantara L.C., Angeletti S., Ciccozzi M. (2020). The global spread of 2019-nCoV: A molecular evolutionary analysis. Pathog. Glob. Health.

[12] Pascarella S., Ciccozzi M., Benvenuto D., Borsetti A., Cauda R., Cassone A. (2022). Peculiar Variations of the Electrostatic Potential of Spike Protein N-terminal Domain Associated with the Emergence of Successive SARS-CoV-2 Omicron Lineages. J. Infect..

[13] Grifoni A., Weiskopf D., Ramirez S.I., Mateus J., Dan J.M., Moderbacher C.R., Rawlings S.A., Sutherland A., Premkumar L., Jadi R.S. (2020). Targets of T Cell Responses to SARS-CoV-2 Coronavirus in Humans with COVID-19 Disease and Unexposed Individuals. Cell.

[14] De la Fuente I.M., Malaina I., Fedetz M., Chruszcz M., Grandes G., Targoni O., Lozano-Perez A.A., Shteyer E., Ya’akov A.B., de la Camara A.G. (2022). Stability of SARS-CoV-2 spike antigens against mutations. medRxiv.

[15] Greaney A.J., Starr T.N., Bloom J.D. (2022). An antibody-escape estimator for mutations to the SARS-CoV-2 receptor-binding domain. Virus Evol..

[16] Gruell H., Vanshylla K., Tober-Lau P., Hillus D., Sander L.E., Kurth F., Klein F. (2022). Neutralisation sensitivity of the SARS-CoV-2 omicron BA.2.75 sublineage. Lancet Infect..

[17] Wang L., Cheng G. (2022). Sequence analysis of the emerging SARS-CoV-2 variant Omicron in South Africa. J. Med. Virol..

[18] Liu L., Iketani S., Guo Y., Chan J.F.-W., Wang M., Liu L., Luo Y., Chu H., Huang Y., Nair M.S. (2022). Striking antibody evasion manifested by the Omicron variant of SARS-CoV-2. Nature.

[19] Moriyama M., Hugentobler W.J., Iwasaki A. (2020). Seasonality of Respiratory Viral Infections. Annu. Rev. Virol..

[20] Katoh K., Standley D.M. (2013). MAFFT Multiple sequence alignment software version 7: Improvements in performance and usability. Mol. Biol. Evol..

[21] Okonechnikov K., Golosova O., Fursov M. (2012). UGENE Team. Unipro UGENE: A unified bioinformatics toolkit. Bioinformatics.

[22] Darriba D., Taboada G.L., Doallo R., Posada D. (2012). jModelTest 2: More models, new heuristics and parallel computing. Nat. Methods.

[23] Drummond A.J., Rambaut A. (2007). BEAST: Bayesian evolutionary analysis by sampling trees. BMC Evol. Biol..

[24] Kass R.E., Raftery A.E. (1995). Bayes factors. J. Am. Stat. Assoc..

[25] Mugosa B., Vujosevic D., Ciccozzi M., Valli M.B., Capobianchi M.R., Presti A.L., Cella E., Giovanetti M., Lai A., Angeletti S. (2016). Genetic diversity of the haemagglutinin (HA) of human influenza A (H1N1) virus in Montenegro: Focus on its origin and evolution. J. Med. Virol..

[26] Webb B., Sali A. (2017). Protein structure modeling with MODELLER. Methods Mol. Biol..

[27] Schrodinger L.L.C. (2015). The PyMOL Molecular Graphics System.

[28] Olsson M.H.M., Søndergaard C.R., Rostkowski M., Jensen J.H. (2011). PROPKA3: Consistent treatment of internal and surface residues in empirical p K a predictions. J. Chem. Theory Comput..

[29] Delgado J., Radusky L.G., Cianferoni D., Serrano L. (2019). FoldX 5.0: Working with RNA, small molecules and a new graphical interface. Bioinformatics.

[30] Krüger D.M., Gohlke H. (2010). DrugScorePPI webserver: Fast and accurate in silico alanine scanning for scoring protein-protein interactions. Nucleic Acids Res..

[31] Kringelum J.V., Lundegaard C., Lund O., Nielsen M. (2012). Reliable B Cell Epitope Predictions: Impacts of Method Development and Improved Benchmarking. PLoS Comput. Biol..

