# Supplementary material for: Genetic and Structural Data on the SARS-CoV-2 Omicron BQ.1 Variant Reveal Its Low Potential for Epidemiological Expansion
Source: Int J Mol Sci. 2022 Dec 3;23(23):15264. doi: 10.3390/ijms232315264 (PMC9739521; doi:10.3390/ijms232315264)
Supplement: Supplementary file 1 [file ijms-23-15264-s001.zip › Table S1.pdf]

SUPPLEMENTAL TABLE

Data Availability

GISAID Identifier:	EPI_SET_221108qe
doi:	10.55876/gis8.221108qe

All genome sequences and associated metadata in this dataset are published in GISAID's EpiCoV database. To view the contributors of each individual sequence with details such as accession number, Virus name, Collection date, Originating Lab and Submitting Lab and the list of Authors, visit 10.55876/gis8.221108qe

Data Snapshot

- EPI_SET_221108qe is composed of 1,114 individual genome sequences.
- The collection dates range from 2022-07-10 to 2022-10-05;
- Data were collected in 35 countries and territories;
- All sequences in this dataset are compared relative to hCoV-19/Wuhan/WIV04/2019 (WIV04), the official reference sequence employed by GISAID (EPI_ISL_402124). Learn more at https://gisaid.org/WIV04.

SUPPLEMENTAL TABLE

Data Availability

GISAID Identifier: EPI_SET_221109dq
doi: 10.55876/gis8.221109dq

All genome sequences and associated metadata in this dataset are published in GISAID's EpiCoV database. To view the contributors of each individual sequence with details such as accession number, Virus name, Collection date, Originating Lab and Submitting Lab and the list of Authors, visit 10.55876/gis8.221109dq

Data Snapshot

- EPI_SET_221109dq is composed of 91 individual genome sequences.
- The collection dates range from 2022-07-01 to 2022-09-19;
- Data were collected in 25 countries and territories;
- All sequences in this dataset are compared relative to hCoV-19/Wuhan/WIV04/2019 (WIV04), the official reference sequence employed by GISAID (EPI_ISL_402124). Learn more at https://gisaid.org/WIV04.

SUPPLEMENTAL TABLE

Data Availability

GISAID Identifier:	EPI_SET_221109fb
doi:	10.55876/gis8.221109fb

All genome sequences and associated metadata in this dataset are published in GISAID's EpiCoV database. To view the contributors of each individual sequence with details such as accession number, Virus name, Collection date, Originating Lab and Submitting Lab and the list of Authors, visit 10.55876/gis8.221109fb

Data Snapshot

- EPI_SET_221109fb is composed of 92 individual genome sequences.
- The collection dates range from 2022-08-21 to 2022-10-07;
- Data were collected in 22 countries and territories;
- All sequences in this dataset are compared relative to hCoV-19/Wuhan/WIV04/2019 (WIV04), the official reference sequence employed by GISAID (EPI_ISL_402124). Learn more at https://gisaid.org/WIV04.

SUPPLEMENTAL TABLE

Data Availability

GISAID Identifier:	EPI_SET_221109kc
doi:	10.55876/gis8.221109kc

All genome sequences and associated metadata in this dataset are published in GISAID's EpiCoV database. To view the contributors of each individual sequence with details such as accession number, Virus name, Collection date, Originating Lab and Submitting Lab and the list of Authors, visit 10.55876/gis8.221109kc

Data Snapshot

- EPI_SET_221109kc is composed of 94 individual genome sequences.
- The collection dates range from 2022-08-09 to 2022-09-26;
- Data were collected in 8 countries and territories;
- All sequences in this dataset are compared relative to hCoV-19/Wuhan/WIV04/2019 (WIV04), the official reference sequence employed by GISAID (EPI_ISL_402124). Learn more at https://gisaid.org/WIV04.

SUPPLEMENTAL TABLE

Data Availability

GISAID Identifier: EPI_SET_221109vo
doi: 10.55876/gis8.221109vo

All genome sequences and associated metadata in this dataset are published in GISAID's EpiCoV database. To view the contributors of each individual sequence with details such as accession number, Virus name, Collection date, Originating Lab and Submitting Lab and the list of Authors, visit 10.55876/gis8.221109vo

Data Snapshot

- EPI_SET_221109vo is composed of 98 individual genome sequences.
- The collection dates range from 2022-07-21 to 2022-09-29;
- Data were collected in 16 countries and territories;
- All sequences in this dataset are compared relative to hCoV-19/Wuhan/WIV04/2019 (WIV04), the official reference sequence employed by GISAID (EPI_ISL_402124). Learn more at https://gisaid.org/WIV04.

SUPPLEMENTAL TABLE

Data Availability

GISAID Identifier:	EPI_SET_221109om
doi:	10.55876/gis8.221109om

All genome sequences and associated metadata in this dataset are published in GISAID's EpiCoV database. To view the contributors of each individual sequence with details such as accession number, Virus name, Collection date, Originating Lab and Submitting Lab and the list of Authors, visit 10.55876/gis8.221109om

Data Snapshot

- EPI_SET_221109om is composed of 86 individual genome sequences.
- The collection dates range from 2022-09-09 to 2022-10-05;
- Data were collected in 17 countries and territories;
- All sequences in this dataset are compared relative to hCoV-19/Wuhan/WIV04/2019 (WIV04), the official reference sequence employed by GISAID (EPI_ISL_402124). Learn more at https://gisaid.org/WIV04.
