# Supplementary material for: Genetic and Structural Data on the SARS-CoV-2 Omicron BQ.1 Variant Reveal Its Low Potential for Epidemiological Expansion
Source: Int J Mol Sci. 2022 Dec 3;23(23):15264. doi: 10.3390/ijms232315264 (PMC9739521; doi:10.3390/ijms232315264)
Supplement: Supplementary file 1 [file ijms-23-15264-s001.zip › Table S2.pdf]

Supplementary Table S2

Comparison of the BA.5 and BQ.1 mutations in the NTD and RBD regions of the Spike

	**BA.5**	**BQ.1**
NTD	T9I	T9I
	L24S	L24S
	Δ25-27	Δ25-27
	Δ69-70	Δ69-70
	G142D	G142D
	V213G	V213G
RBD	G339D	G339D
		R346T
	S371F	S371F
	S373P	S373P
	S375F	S375F
	T376A	T376A
	D405N	D405N
	R408S	R408S
	K417N	K417N
	N440K	N440K
		K444T
	L452R	L452R
		N460K
	S477N	S477N
	T478K	T478K
	E484A	E484A
	F486V	F486V
	Q498R	Q498R
	N501Y	N501Y
	Y505H	Y505H
