# Supplementary figures and images for: Genetic and Structural Data on the SARS-CoV-2 Omicron BQ.1 Variant Reveal Its Low Potential for Epidemiological Expansion
Source: Int J Mol Sci. 2022 Dec 3;23(23):15264. doi: 10.3390/ijms232315264 (PMC9739521; doi:10.3390/ijms232315264)

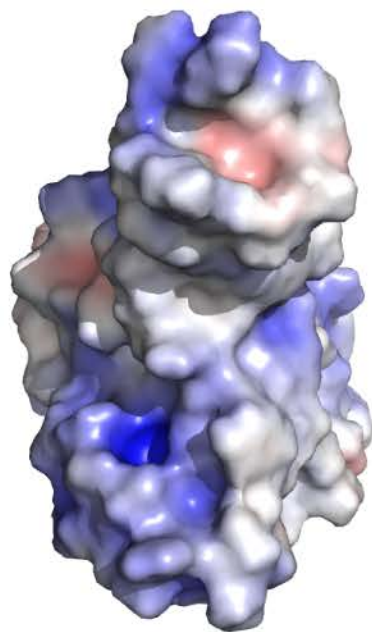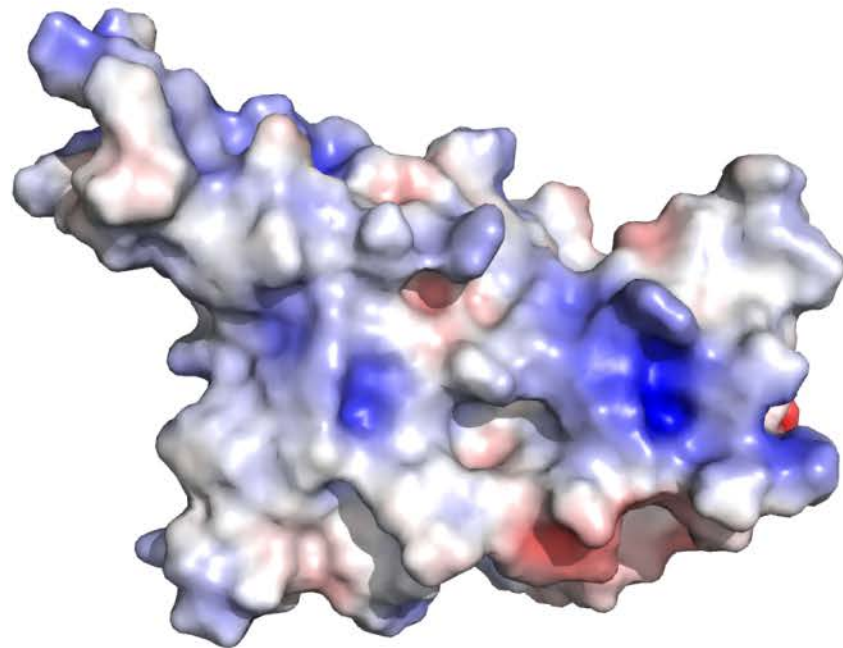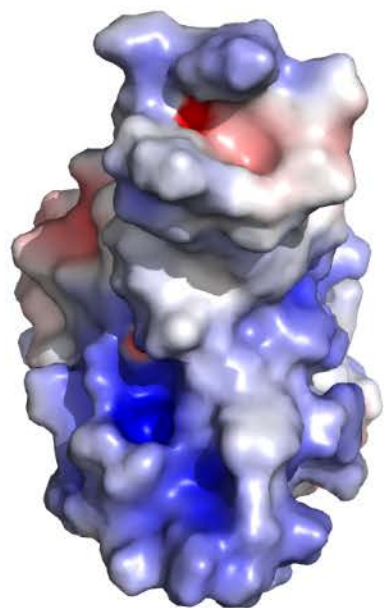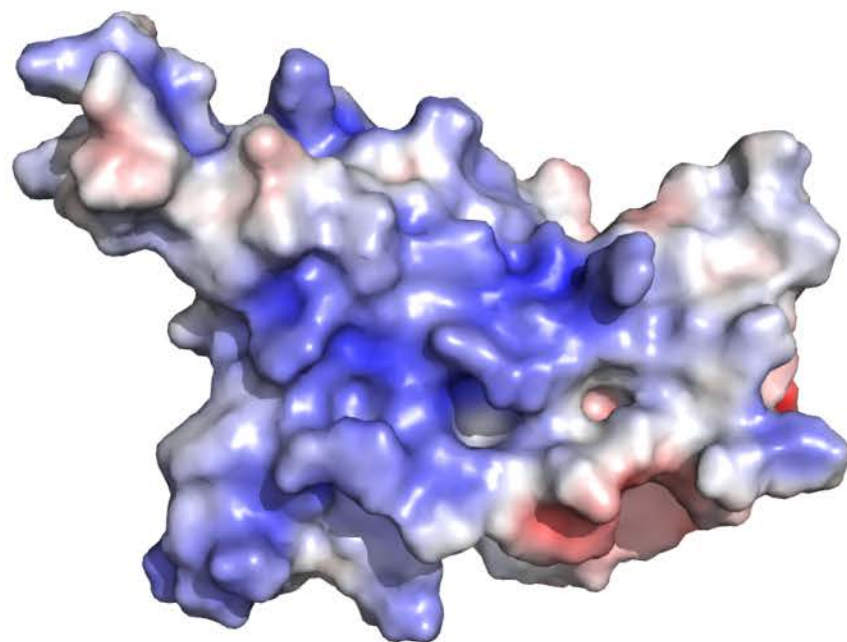

**A**

**B**

Supplement: Supplementary file 1 [file ijms-23-15264-s001.zip › Figure S1.pdf]
